# Flecainide Toxicity Presenting as Wide-Complex Tachycardia in a Patient with Refractory Atrial Fibrillation

**DOI:** 10.51894/001c.165585

**Published:** 2026-07-31

**Authors:** Anthony Costa, Mitchell Rasak, Sydney Moriarty, Sabrina Bahu, Albert Alvarado, Thomas Vanhecke

**Affiliations:** 1 Henry Ford - Genesys

## 75

### Background

Classic antiarrhythmics such as flecainide are effective for AF rhythm control but have a narrow therapeutic window. Toxicity may mimic ventricular tachycardia with conduction delay.

### Case DESCRIPTION

A 67-year-old woman with persistent atrial fibrillation refractory to three ablations, HFpEF, pulmonary hypertension, hypertension, and obstructive sleep apnea presented for elective cardioversion. She was found to have wide-complex tachycardia with an irregular rhythm (QRS 213 ms, baseline 157 ms; QTc 619 ms). One month earlier, her flecainide dose was increased from 50 mg BID to 100 mg BID, taken intermittently for palpitations. She reported exertional dyspnea and palpitations but denied chest pain or syncope. Labs showed intact renal/hepatic function and mild hypokalemia. Flecainide level was therapeutic at 0.54 µg/mL, yet toxicity was suspected given the marked conduction delay. Initially suspected VT, but irregular rhythm, recent dose escalation, and context favored flecainide toxicity.

### Decision Making

She was admitted to the MICU. Flecainide was discontinued and poison control advised IV sodium bicarbonate titrated to alkalemia (pH 7.45–7.55) to mitigate sodium channel blockade, with electrolyte repletion targeting high-normal potassium, magnesium, and phosphorus. Serial ECGs demonstrated QRS/QTc narrowing. Anticoagulation with rivaroxaban was maintained and metoprolol was carefully resumed for rate control, balancing risk of worsening conduction delay. She remained hemodynamically stable without ventricular arrhythmias. Conduction normalized over several days. She was discharged on beta-blocker therapy, with outpatient follow-up emphasizing alternatives to Class Ic agents, including ablate-and-pace for recurrent AF and antiarrhythmic failure.

### Conclusion

This case highlights flecainide toxicity as a reversible cause of wide-complex tachycardia, even at therapeutic levels. Early recognition, drug discontinuation, sodium bicarbonate therapy, and electrolyte optimization are key to preventing life-threatening arrhythmias.

